# Supplementary material for: Polyamine Transport Protein PotD Protects Mice against Haemophilus parasuis and Elevates the Secretion of Pro-Inflammatory Cytokines of Macrophage via JNK–MAPK and NF–κB Signal Pathways through TLR4
Source: Vaccines (Basel). 2019 Dec 14;7(4):216. doi: 10.3390/vaccines7040216 (PMC6963478; doi:10.3390/vaccines7040216)
Supplement: Supplementary file 1 [file vaccines-07-00216-s001.pdf]

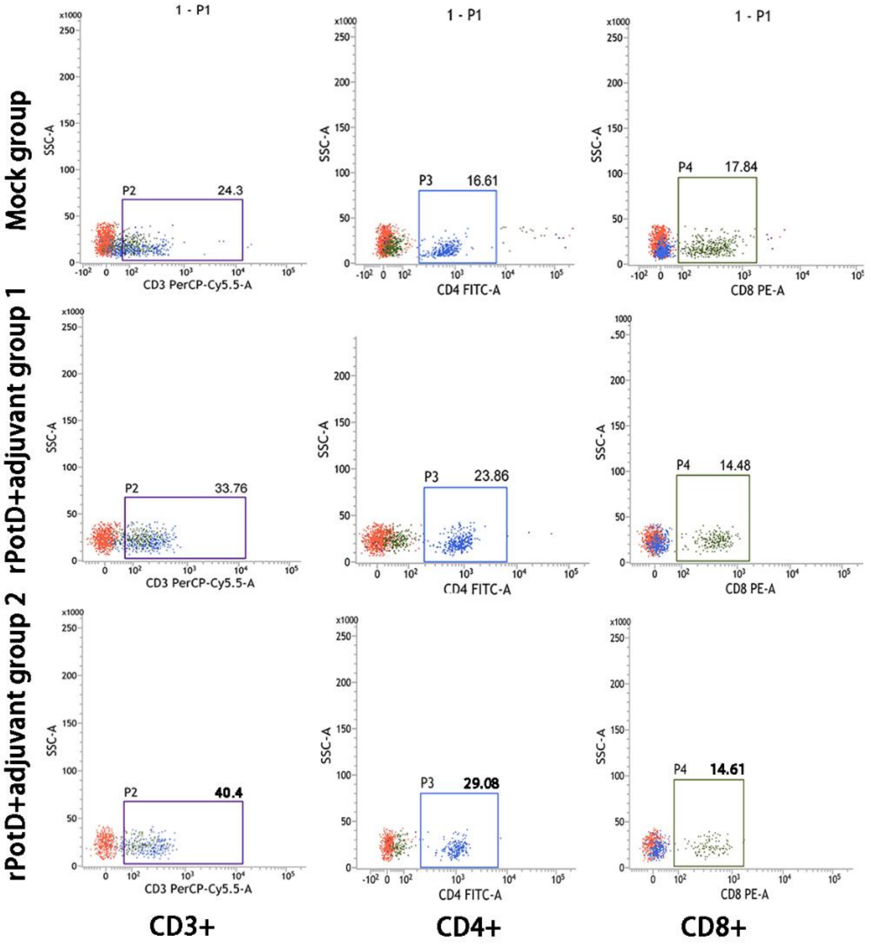

Figure S1. Percentages of total T cells (mature T lymphocytes) and the T cell subsets T-helper cells (CD4+) and T suppressors/cytotoxic T-cells (CD8+) in a representative peripheral blood sample by FCM (%). All experiments were performed in triplicate with at least 10,000 cells per treatment. The first lane: CD3+. The second lane: CD4+. The third lane: CD8+. The first row: Mock group. The second row: rPotD+adjuvant group 1. The third row: rPotD+adjuvant group 2.

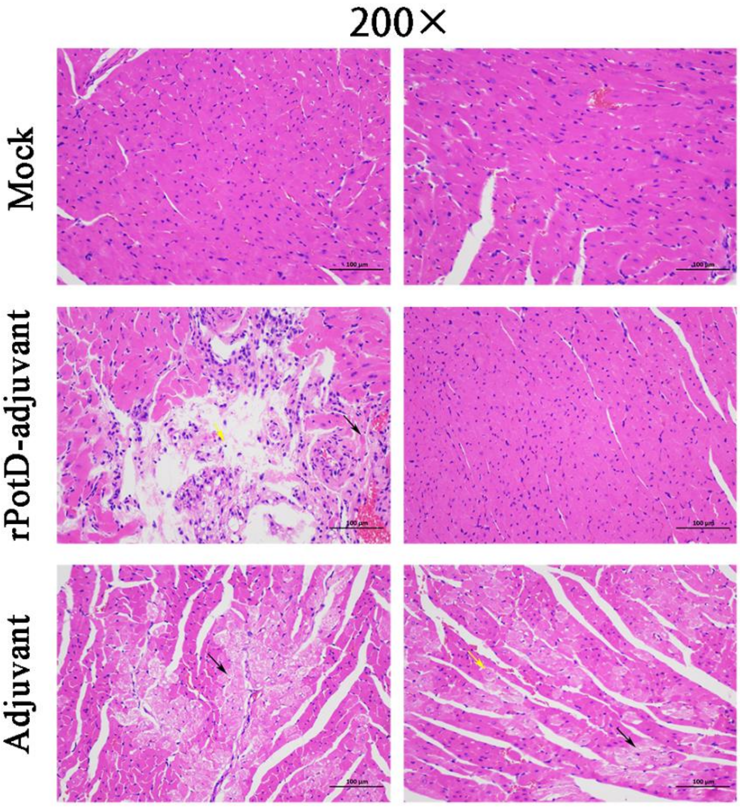
200×
Mock
rPotD-adjuvant
Adjuvant

(A)

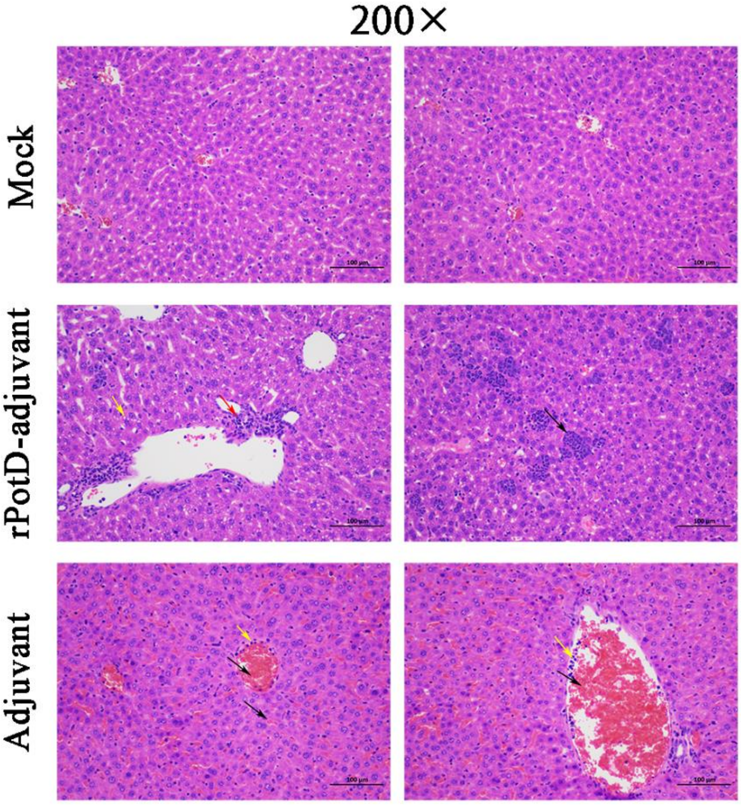
200×
Mock
rPotD-adjuvant
Adjuvant

(B)

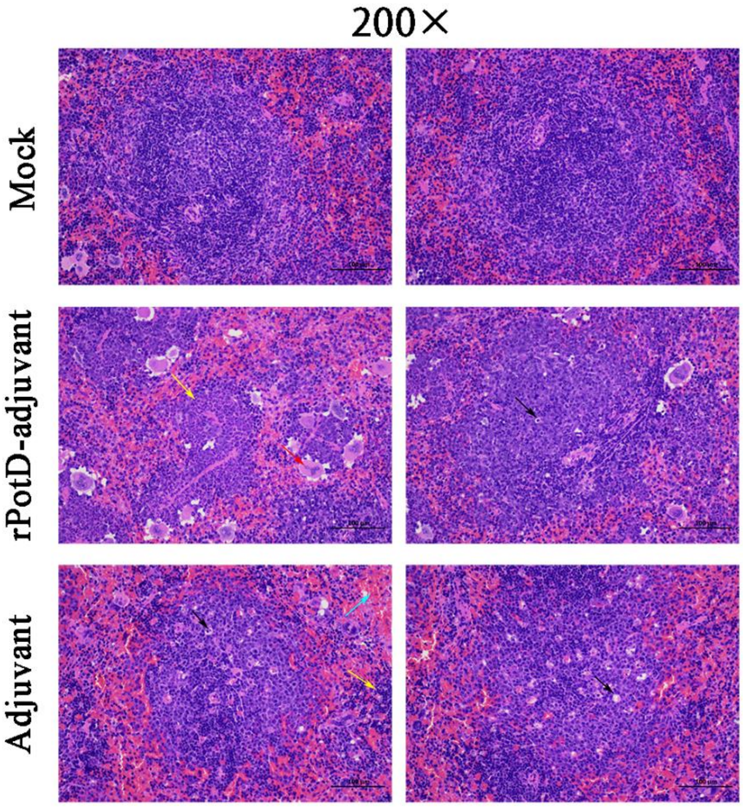

(C)

Figure S2. Histopathologic analysis of mice hearts, livers and spleens (200×). Hearts, livers and spleens tissues were harvested from mice in different groups after 72 h dpi, and used for HE staining and histopathological assay. (A) Hearts. (B) Livers. (**C) Spleens. Obvious** lesions have been marked with arrows. Main pathological changes in wild-type SC1401 and complemented groups: **(Hearts)** myocardial fiber necrosis, nuclear pyknosis, deep staining or dissolution; neutrephil infiltration. **(Livers)** A large number of congestions and dilations in central veins, portal veins and hepatic sinus; a multitude of red blood cells can be seen in the venous cava and hepatic sinus (black arrow); adhesive white blood cells are widespread in central veins and portal veins (yellow arrow). **(Spleens)** lymphocytic necrosis, hyperchromatism or dissolution in splenic nodule (black arrow); more extramedullary hematopoietic cells appear in the red pulps (yellow arrow), and the splenic sinus is dilated with congestion (blue arrow).

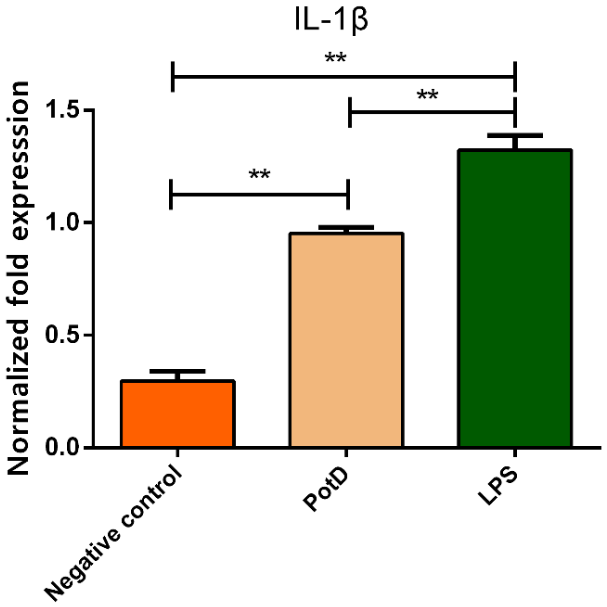
IL-1β
**
**
**
Normalized fold expression
1.5
1.0
0.5
0.0
Negative control
PotD
LPS

(A)

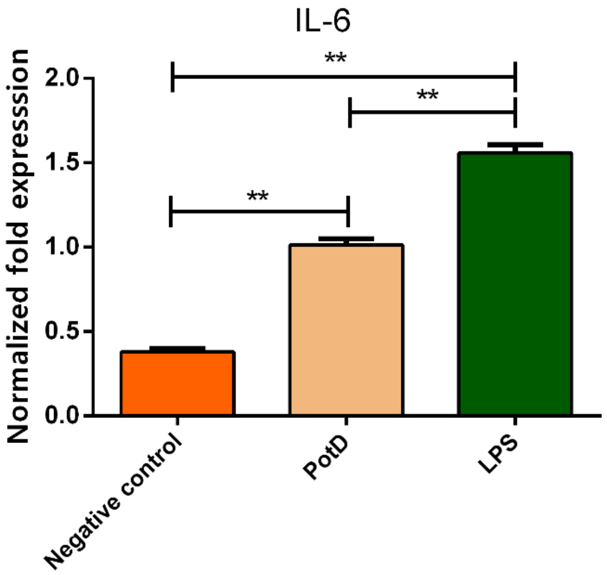
IL-6
**
**
**
Normalized fold expression
2.0
1.5
1.0
0.5
0.0
Negative control
PotD
LPS

(B)

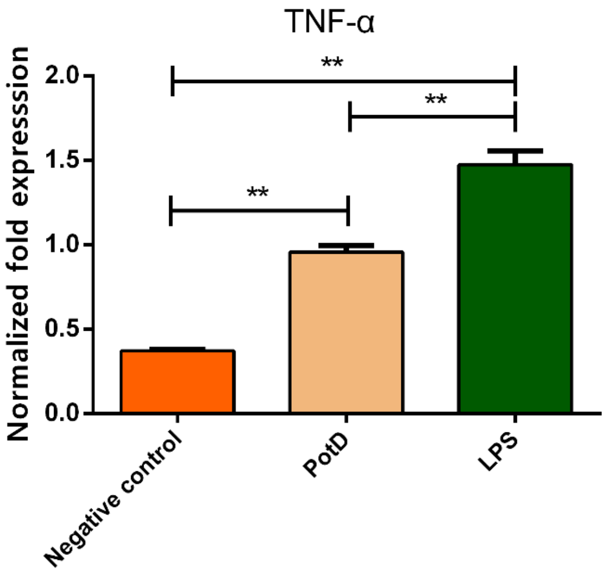

(C)

Figure S3. qPCR detection of the levels of pro-inflammatory cytokines induced by rPotD. Induction of cytokines in Raw 264.7 macrophages by rPotD stimulation. Raw 264.7 macrophages were incubated with 15 μg/mL rPotD and 200 ng/mL LPS (positive control) for 12 h, as well as sole culture media (negative control). Quantification analysis was conducted to analyze the transcriptional levels of IL-1β **(A)**, IL-6 **(B)**, TNF-*α* **(C)**. GAPDH was chosen to be a reference gene in relatively quantification analysis.

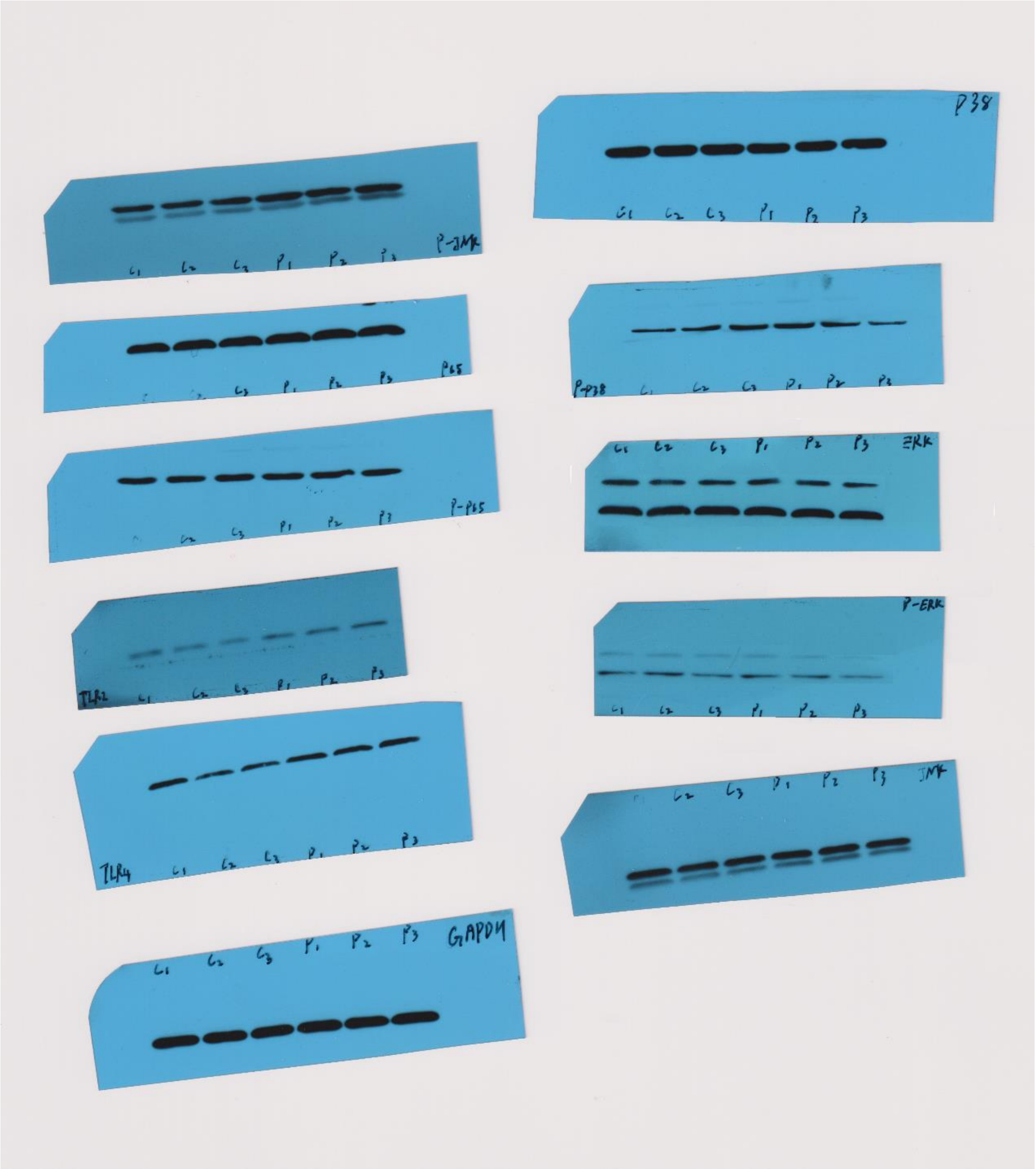

Figure 4. The raw data of western blotting used in this study.

original data:

gray value:

	mock	mock	mock	PotD	PotD	PotD
P38/GAPDH	1.104373	1.118056	1.091653	1.028157	0.9923858	0.9989956
P-P38/GAPDH	0.3185404	0.4164839	0.4640844	0.4580914	0.3627991	0.2496564
ERK/GAPDH	0.4282264	0.3737208	0.380251	0.342344	0.3676577	0.3099383
P-ERK/GAPDH	0.8035775	0.8120614	0.8390253	0.7870283	0.7975345	0.7409339

JNK/GAPDH	0.1845989	0.2072368	0.1849427	0.1965893	0.1856418	0.1656388
P-JNK/GAPDH	0.6797245	0.7163743	0.7855974	1.05225	1.070522	1.128634
P65/GAPDH	0.8121277	0.8245614	0.8444445	0.8996372	0.8962654	0.9016387
P-P65/GAPDH	0.5076469	0.5250.5581015	0.5930697	0.6054387	0.5634537	
TLR2/GAPDH	0.1176997	0.1111111	0.1057647	0.1258708	0.1357324	0.141674
TLR4/GAPDH	0.3553171	0.3108553	0.3312602	0.4571843	0.4650472	0.4757709

ratio：

P-P38/P38	0.2884354	0.3725074	0.4251208	0.4455463	0.3655828	0.2499074
P-ERK/ERK	1.876525	2.172909	2.206504	2.29894	2.169231	2.390585
P-JNK/JNK	3.682171	3.45679	4.247787	5.352529	5.766602	6.81383
P-P65/P65	0.6250826	0.6367021	0.6609097	0.6592321	0.6755127	0.6249219

Figure S4. The raw data of western blotting used in this study.
